# Evidence-based art of the clinical examination

**DOI:** 10.1590/S1516-31802000000100001

**Published:** 2000-01-02

There is a spurious discussion about Evidence Based Medicine not being "the true medicine", made at the bedside. This is a misunderstanding, made and used by poorly informed, so to say, low-grade medical practitioners. Two kinds of information are needed for the clinical decision: the information based on the best clinical research and the information based on the patient (interview and physical examination).^1^ One without the other makes no sense, but both together, with good quality, are fundamental in clinical practice. Empirical evidence of precision and accuracy of the clinical examination is used daily in clinical assistance. However, "real" precision and accuracy of the clinical examination is needed.^2^ The interest in this subject, world-wide, is increasing. The Journal of the American Medical Association (JAMA) has been publishing, since 1992, a series of overviews on the rational clinical examination;^3^ in a section named The Rational Clinical Examination, there are other good examples available on the Internet.^4^

CARE study [the Clinical Assessment of the Reliability of the Examination (CARE) – A Proposal for Collaborative Studies of the Accuracy and Precision of the Clinical Examination]. A group now working at the NHS R&D Centre for Evidence-Based Medicine in Oxford is trying to solve the problems of both numbers and clinical applicability by catalyzing the execution of large (>100 clinicians enrolling >1000 patients), simple (<2 minutes per patient and <15 patients per participating clinician), fast (<2 weeks, with automatic data entry via the Internet) studies of the accuracy and precision of specific elements of the history and physical examination. Their initial efforts led to >160 clinicians from 20 countries joining CARE. CARE works like this: a) Anybody, at any stage of training or experience, can join the enterprise just by signing up for it. The only pre-requisites are an interest in the clinical examination, access to the Internet, and a well-developed sense of humour. b) Individuals in the collaboration nominate symptoms and signs they would like to validate (or not) and broadcast them to the membership. c) Members who share an interest in this same topic come together electronically as Investigators, and proceed to design and debug the protocol and offer it to the entire collaboration. d) The membership-at-large vote with their precious time, enrolling just a few patients each and reporting their results electronically. e) Analyses are shared, PowerPoint summaries posted, and papers published (with authorship by the Investigators, on behalf of CARE, and acknowledging every member who entered the requisite number of patients). URL: http://www.carestudy.com

Clinical Examination Research Interest Group of the Society of General Internal Medicine. It is possible to find clinical information about the physical examination: a) Rational Clinical Examination series. b) Search PubMed and Bedside Diagnosis for information about the value of clinical findings. c) Ongoing research about the physical examination. d) Studies of the physical examination performed by the Clinical Examination Research Interest Group; e) Links to CARE study. URL: http://www.sgim.org/interestgroups/clinexam.html

Another website is owned by the American College of Physicians, the Bedside Diagnosis, containing an annotated bibliography of literature on physical examination and interviewing. URL: http://www.acponline.org/public/bedside/index.html

In a short time, these tasks will probably have generated information (evidence) that will cause a deep impact on patient diagnosis and care. And, the final step, the patient-physician relation will be improved. The optimal model of physician is one who has three components: a) expertise the medical interviewing / physical examination; b) clinical epidemiology / quantitative clinical reasoning; c) Ethical physician-patients interaction. Alea jacta est!
